# Mapping renewable energy futures in the Cologne planning region: Land-use constraints and landscape impacts

**DOI:** 10.1007/s00267-026-02474-5

**Published:** 2026-04-30

**Authors:** Dragan Petrovic, Friederike Schrade, Stephan Bosch, Harald Kunstmann

**Affiliations:** 1 University of Augsburg, Institute of Geography, Augsburg, Germany; 2 University of Augsburg, Centre for Climate Resilience, Augsburg, Germany; 3 Institute of Meteorology and Climate Research, Karlsruhe Institute of Technology, Campus Alpin, Garmisch-Partenkirchen, Germany

**Keywords:** Regional sustainability, Energy transition, Renewable energy siting, Energy landscapes, Spatial planning, Land use scenarios

## Abstract

Germany aims to become climate-neutral by 2045, which necessitates a far-reaching transformation of its energy system. Central to this shift is the ongoing expansion of renewable energy sources, particularly wind power and photovoltaics. This study seeks to model the growth of renewable energy systems in the Cologne planning region in western Germany in a way that is consistent with national and international climate targets. For this purpose, we employ a Geographic Information System (GIS)-based methodology combined with a siting algorithm. This allows us to apply land-use regulation scenarios to simulate various regulatory pathways and evaluate their effects on the resulting energy landscapes. The modeling period spans 2023–2045, and population data, CO₂ budgets, and electricity demand are derived for this timeframe. The findings indicate that climate-neutral electricity production in the region cannot be achieved under the current planning framework due to a lack of sufficient land for renewable energy installations. Even in scenarios with more flexible planning rules and higher-efficiency wind turbines, available land remains scarce. Only a substantial reduction in energy demand, modeled in this study at 50%, would make the targets attainable. Regardless, the energy transition would profoundly reshape the landscape, as land requirements are substantial in every scenario. Nonetheless, the region could still markedly increase the share of renewable electricity production through targeted measures. The study confirms that combining a GIS-based approach with a siting algorithm is a robust method for exploring alternative energy pathways.

## Introduction

Germany aims to be climate-neutral by 2050, but current efforts to expand renewable energy (RE) are insufficient. Meeting CO₂ reduction targets would require accelerating RE expansion by a factor of three to seven (UBA 2024a). In a densely populated country like Germany, limited land and various spatial restrictions such as nature protection and monument conservation makes the attainment of this goal uncertain (Bosch 2021).

Data on the availability of land for the energy transition, which includes both areas currently permitted by law and potential future sites, is scarce. Although the Reiner Lemoine Institute’s *PV and Wind calculator* indicates sufficient land potential in Germany (Saerbeck et al. 2021), the designation of sites remains limited due to the implementation and interpretation of legal frameworks, as well as the way political actors balance competing interests. An explicit method for optimally locating RE systems that complies with the Paris Agreement and existing planning regulations is still missing.

Identifying suitable, low-conflict sites for RE is essential but highly complex. Site selection often requires balancing competing land uses such as species conservation, forestry, agriculture, settlements (e.g., Dale et al. 2011; Armstrong et al. 2014; Bulling 2016; de Magalhães et al. 2019; Straka et al. 2020; Tafarte and Lehmann 2021) and the degree of compromise varies with local social and ecological conditions. In some areas, for instance, conflicts between species protection and RE are more pronounced than in others (Schuster et al. 2015; Bulling 2016; Tanaka et al. 2022).

Geographic information system (GIS)-based methods are widely used to identify suitable areas for wind and PV deployment, typically through multi-criteria analyses that assess land availability, grid access, environmental and technical constraints, meteorological conditions and socio-economic factors. Jbaihi et al. (2022) and Fakharizadehshirazi and Rösch (2024) offer overviews of the constraining criteria. Common weighting methods include Network Analysis Process (NAP), the Technique for Order of Preference by Similarity to Ideal Solution (TOPSIS), and especially the Analytic Hierarchy Process (AHP), which dominates recent research (e.g., Höfer et al. 2016; Saraswat et al. 2021; Shorabeh et al. 2022a and Fakharizadehshirazi and Rösch 2024). These approaches have been applied globally and across all spatial scales, from regional wind studies in Germany (Höfer et al. 2016), Iran (Shorabeh et al. 2022b), and Iraq (Nassif et al. 2024) to PV assessments in Morocco (Jbaihi et al. 2022), China (Cui et al. 2023), Spain (Luis-Ruiz et al. 2024), and Germany (Fakharizadehshirazi and Rösch 2024), as well as combined wind–-solar analyses in India (Saraswat et al. 2021), Africa (Doorga et al. 2022) and Germany (Weber et al. 2023). Other GIS-based tools without multi-criteria components include GOMap (McGhee 2023) and regional suitability assessments for Poland (Benalcazar et al. 2024) and Nigeria (Okedu et al. 2024). However, most studies focus only on mapping current suitability rather than simulating how RE deployment could evolve over time under planning rules, energy needs, CO₂ limits, or socio-spatial trade-offs. An exception is Bosch and Kienmoser (2024), who model scenario-based changes to regional planning guidelines for Germany’s Augsburg region through 2045. There are also multi-criteria approaches that do not involve GIS. For example, Weber (2023) used a multi-criteria scenario framework to analyze trade-offs between RE expansion, biodiversity, and land use at the regional level. This analysis was based on stakeholder surveys and a German case study.

Several studies have explored RE site deployment in Germany. Jung et al. (2018) modeled wind turbine siting scenarios to reach a 40% wind share, varying distribution criteria, administrative roles, repowering, and turbine types using a national 200 m × 200 m grid. Building on earlier work on setback rules and designated areas by Lauf et al. (2020) and Stede and May (2020), Meier et al. (2024) examined how spatial planning policies affected wind expansion from 2000 to 2016. However, these studies do not assess how deviations from current planning laws – likely necessary for future land use – might enable or constrain a climate-neutral energy system. Nor do they clarify the social and environmental impacts of such spatial changes. None provides a model showing how wind and PV sites would evolve over time under shifting planning rules, energy needs, and CO₂ budgets.

Therefore, the aim of this study is to model the expansion of wind and PV systems in the Cologne administrative district in a way that meets national and international climate protection goals. Using a GIS-based approach, the study aligns regional energy planning with Germany’s carbon neutrality target of 2045 while ensuring that the remaining global CO₂ budgets under the two-degree limit are proportionally respected. We derive the following research questions from this objective:

1. How can regional (renewable) energy landscapes be modeled within spatial planning constraints to achieve climate-neutral electricity production in line with the Paris climate goals?
2. To what extent do current planning regulations impede the realization of a climate-neutral electricity sector?
3. What socio-technical implications accompany changes to the landscape?
4. How would changes in planning law affect the availability of land for renewable energy projects?

The study first analyzes how current planning regulations enable or limit RE deployment, identifying both technically overdeveloped areas and those restricted by legal constraints. It then tests alternative scenarios by adjusting planning rules, for example, easing competing land-use restrictions or redistributing landscape burdens. Finally, by incorporating regional CO₂ budgets, energy demand, and technological developments, the study models annual RE deployment across multiple policy pathways. This approach highlights how changing planning laws and emerging technologies can shape future energy landscapes. It assumes that the current spatial planning regime still reflects the restrictive land use logic of fossil fuel-based industrial societies, where energy production required little land or was spatially externalized and therefore treated as secondary to competing land uses such as nature conservation, recreation, and settlements. In light of the ecological crisis, geopolitical risks, and climate neutrality goals, this outdated framework limits the state’s ability to act. For this reason, in addition to a baseline scenario based on current planning law, we explore alternative spatial futures by varying legal requirements for nature protection, species conservation, and distances to settlements. We also consider expanding REs into regions that do not yet meet the requirements for the spatial implementation of energy technologies aiming at increasing social justice. These variations give the opportunity to highlight where planning decisions have the greatest impact on feasibility and where current frameworks may underestimate the spatial demands of decarbonization. Rather than proposing a concrete planning framework for RE expansion, our spatial scenarios aim to stimulate debates about a paradigm shift in the provision of land for REs. To analyze the technological resilience of planning regions, we have raised the spatial endurance test to the maximum level of a politically targeted energy transition. However, regional demand for RE may be below this maximum if energy is imported from neighboring regions or if regional energy demand cannot be met by the region’s own land. Less densely populated regions may therefore contribute disproportionately to national climate goals. In any case, due to the numerous land interests and competitions, the Cologne planning region is an enlightening case study for developing a climate-neutral energy supply. It offers a blueprint for industrial regions worldwide whose planning regimes have not yet been adapted to climate protection requirements.

Against this backdrop, our study introduces three key innovations: (1) siting RE in alignment with the Paris Agreement within a densely populated region; (2) the integration of a GIS-based approach with a detailed siting algorithm; and (3) the exploration of alternative planning pathways. By incorporating concrete climate targets into the modeling process, our approach ensures that RE expansion is strategically aligned with policy objectives. The alternative planning pathways allow for a systematic analysis of how varying priorities such as distributional justice, nature conservation, distance constraints, technological progress, and electricity demand shape the spatial patterns of the energy transition. Unlike Bosch and Kienmoser (2024), who rely on aggregated wind energy zones, our siting algorithm places individual wind turbines with exact dimensions and spacing requirements, enabling a higher degree of spatial accuracy. Given the availability of appropriate input data, this approach is adaptable to other regions and contexts.

## Methodology

This section outlines our methodological approach. We first describe the study region, followed by a description of how we derived the core data inputs: (regional) CO₂ budgets, population data, and electricity demand projections. We then explain the GIS-based steps used to generate the spatial data required for the siting algorithm, followed by a detailed overview of the land-use scenarios. Finally, we introduce the siting algorithm itself, an innovative method for allocating RE sites and modeling alternative energy landscape futures. Energy landscapes are understood as the parts of the physical environment shaped by energy transitions, reflecting the evolving relationships among technologies, people, places, and their perceptions (Selman 2010; van der Horst and Vermeylen 2011; Pasqualetti 2013; Calvert et al. 2019).

### Study region

With a population of approximately 4.5 million inhabitants and a population density of 616 inhabitants per square kilometer (as of September 30, 2023), the Cologne planning region is one of the most densely populated regions in the country (Statistisches Landesamt NRW 2023). This high population density intensifies competition between different land use interests, making the region particularly suitable for analyzing spatial trade-offs. At the same time, the region is historically and structurally intertwined with fossil fuel energy, particularly lignite, which has shaped its industrial base, employment patterns, and regional economy for decades. The lignite phase-out, originally planned for 2038, has been brought forward to 2030 (Bundesregierung 2022). Nevertheless, the Rhineland mining area is set to remain an energy region, triggering far-reaching transformation processes (MWIKE NRW 2021). As an important center for trade, services, media, and tourism, the metropolis of Cologne gives the region a strong urban character (Rönz 2024). Additionally, the planning region features significant landscape diversity, ranging from low mountain ranges and extensive forest areas (Eifel, Bergisches Land, Siebengebirge) to the agricultural landscape of the Cologne Bay, former heathlands (e.g., Wahner Heide, Drover Heide), and riverine landscapes alongside the Rhine, Sieg, Ruhr, Wupper, and Erft (Adams et al. 2016). Together, these characteristics make the Cologne planning region a challenging yet highly suitable test case for our study. Its high population density, elevated energy demand, limited land availability, and the resulting conflicts over land use create a complex and challenging context for testing the robustness and applicability of our approach.

The regional level provides a suitable analytical scale for translating national climate objectives into spatially explicit expansion pathways. Although national targets, such as achieving climate neutrality by 2045, are set at the federal level, their implementation requires cross-sectoral transformation, particularly in the electricity sector, where decentralized RE demands significant spatial reorganization. Concrete land use decisions, however, are made at the subnational level, where site selection, land use competition and landscape impacts can be adequately represented. In this regard, the regional level provides an optimal balance between sufficient spatial resolution to capture landscape impacts as well as site-specific constraints, and a scale large enough to support meaningful scenario modeling. The regional planning level is a suitable unit of analysis for translating national transformation goals into spatially explicit expansion paths, systematically evaluating cumulative land use effects, and identifying conflicts between renewable technologies and other land uses. In this context, Gailing et al. (2020) also emphasize the growing importance of the regional level, stating that the “decentralization triggered by the development of renewable energies has led to a growing importance of the regional scale and to new forms of regional energy spaces”. At the same time, the regional level ensures comparability, a uniform legal framework within the region, and a harmonized database, which increases analytical control and scenario comparability. Importantly, the Cologne planning region is treated as an in-depth case study, not a representative sample, given the significant variation in land use, protected areas, and regulatory conditions across regions.

### Determination of CO_2_-budgets and electricity demands

Regional CO₂ budgets set the temporal limits for the energy transition, as emissions from fossil-based electricity continue to draw down the available budget. The total regional CO_2_ budget calculation for the 2023-2045 modeling period is based on the IPCC report (2021). Our modeling approach is based on the IPCC-defined budget value of 900 gigatons (Gt), resulting in a global CO₂ budget of 791 Gt in 2023 when considering CO₂ emissions from 2020 to 2022 (109 Gt) (Friedlingstein et al. 2022; Statista 2024a). Assuming an average world population of 8.764 billion people over the modeling period, derived from world population projections to 2100 (Statista 2024b), this yields a per-capita budget of 90.3 tons of CO₂, which is then scaled to the regional population.

Electricity demand for the study area is calculated by converting Germany’s 2023 gross electricity demand (UBA 2024b) to the region’s population on September 30, 2023 (Statistisches Landesamt NRW 2023) and applying a projected 110% increase by 2045 (Übertragungsnetzbetreiber 2023; Statista Research Department 2025), driven mainly by electrification in industry, transport, and heating, as well as hydrogen use and electromobility (Kemmler et al. 2021; McKinsey and Company 2024; BEE 2025a; Stobbe et al. 2025). RE must supply 100% of electricity by 2045. In 2023, the region starts at 18% (Energieatlas NRW 2024). The model increases renewable shares annually until reaching full supply in 2045, after which no further expansion is assumed. Following national PV strategy (BMWK 2023), future PV demand is assumed to split evenly between rooftop and ground-mounted systems, but only ground-mounted PV is modeled due to its landscape impact. To support grid stability, wind and PV are each assumed to provide 50% of electricity, meaning ground-mounted PV covers 25% of total demand.

### GIS-based restriction analysis

This study applies a GIS-based spatio-temporal modeling approach that is underpinned by a siting algorithm drawing on a set of spatial input data. *ArcGISpro* and a detailed digital landscape model (BVV 2023) were used to identify suitable areas for RE expansion, capturing land use and administrative boundaries. As Risch et al. (2022) pointed out, a digital landscape model is superior to the CORINE Land Cover dataset (Copernicus Programme 2018), which Bosch and Kienmoser (2024) used. A 100-meter resolution grid was created, with cell centers serving as reference points for linking natural site factors such as wind speed and solar radiation to estimate the energy yields of reference plants across the region.

To identify potential expansion areas, we first defined areas of land generally suitable for each technology. Wind power was deemed suitable for use on arable land, grassland, semi-natural areas, and forests with slopes under 30° (Riedl et al. 2020). Ground-mounted PV systems were considered suitable for arable land, grassland, semi-natural areas, and conversion areas with slopes of under 45° and north-facing orientations of under 30° (Badelt et al. 2020). From these technically suitable areas, exclusion zones based on legally defined constraints as of 2023, including necessary setback distances, were removed (see Table 1 and Table 2). The assumed setback distances were derived from relevant laws or existing analyses, including distances for first-order water bodies and standing waters exceeding 1 ha (§ 61 BNatSchG), watercourse buffer strips (§ 38 WHG), seismic stations and stations of the German Meteorological Service (LANUV 2023), railway lines (Riedl et al. 2020), airports (LANUV 2023), and motorways and federal roads (§ 9 FStrG). For existing wind turbines, a uniform setback of four times the rotor diameter was applied, roughly averaging the typical five times in the main wind direction and three times in crosswind directions. Distances for settlement areas (§ 249 BauGB; TA Lärm; Riedl et al. 2020) were set at approximately three times the reference installation height for inner residential areas and twice the height for outer residential areas, with remaining settlement distances based on Riedl et al. (2020). Our analysis is based solely on legally defined constraints that are binding at all planning levels, providing a spatial foundation independent of political negotiations or site-specific planning decisions. By focusing on this hard legal baseline, we deliberately exclude politically negotiated allocation targets and site-specific planning instruments, enabling a transparent assessment of the physical and spatial limits of RE deployment.

**Table 1:** Exclusion criteria considered in the wind turbine modeling

Exclusion criteria considered		Legal source
*Nature conservation*	national parks, legally protected biotopes, protected landscape elements, natural monuments, nature conservation areas	BNatschG(n.d.)
*Water protection*	50 m setback for first-order water bodies & standing waters > 1 ha, 5 m watercourse buffer strips, water protection zones I and II	BNatschG(n.d.); WHG(n.d.)
*Military*	military training areas	*Military use – defense interests*
*Infrastructure*	seismic stations with mandatory setback distances, stations of the German Meteorological Service with 5.000 m setback, railway lines with 150 m setback, commercial airports with 4.000 m setback, other airports with 1.500 m setback, pipelines and cableways with 1 rotor diameter setback, motorways with 40 m setback, federal roads with 20 m setback, existing wind turbines with four-fold rotor diameter setback	FStrG(n.d.); StrWG NRW(n.d.) ; LuftVG(n.d.)
*Forest conservation*	deciduous and mixed forests, natural forest areas, wilderness development areas	MEA NRW (2024); LFoG NRW(N.d.); LNatSchG NRW(n.d.)
*Settlement distances*	inner residential areas with 700 m setback, outer residential areas with 460 m setback, health and recreation areas with 750 m setback, industrial and commercial areas with 300 m setback, sports, leisure and recreation areas with 400 m setback	BauNVO(n.d); BauGB(n.d.); TA Lärm(n.d.)

**Table 2:** Exclusion criteria considered in the ground-mounted PV modeling

Exclusion criteria considered		Legal source
*Nature conservation*	national parks, legally protected biotopes, protected landscape elements, natural monuments, nature conservation areas	BNatSchG(n.d.)
*Water protection*	50 m setback for first-order water bodies & standing waters > 1 ha, buffer strips, water protection zones I and II	BNatSchG(n.d.); WHG(n.d.)
*Military*	military training areas	*Military use – defense interests*

To avoid wind turbines encroaching on exclusion zones or unsuitable land, each exclusion area was expanded by one rotor length of the reference turbine. For ground-mounted PV, suitability zones were instead buffered inward by 50 m (half a grid cell) to keep them from overlapping unsuitable areas, reflecting the fact that wind turbines are modeled as points while PV systems occupy 1-hectare grid cells. The study area was then classified as suitable or unsuitable for each technology, linked to grid-cell centers, and exported for further analysis.

The input dataset for the base scenario includes the following variables in a 100-meter raster format: Projected latitude and longitude coordinates, affiliation to the rural district or municipality (OpenGeodata.NRW 2024), the average (1991–2023) global radiation (DWD 2024a) and average (1981–2010) wind speed at 160 m hub height (DWD 2024b), derived wind and PV energy yields based on the reference systems used for wind turbines (Enercon 2022) and ground-mounted PV systems (Canadian Solar 2022), and indicators of each site’s technical and legal suitability for wind and/or PV under current planning rules.

### Land use scenarios

In developing land-use scenarios, we follow Dewald et al. (2019), who require that modeled energy futures should be plausible, coherent, clearly distinguishable, and understandable for policy and planning. GIS-based analyses also demand an appropriate level of aggregation, detailed enough to be meaningful, yet abstract enough to remain computationally feasible.

We model several scenario types, all with a normative foundation based on national and international climate targets (Börjeson et al. 2006). The first scenario assumes that the current legal, political, and spatial framework remains unchanged and extrapolates existing trends. The remaining scenarios represent alternative futures not currently allowed by planning law but potentially necessary given ongoing societal debates about the energy transition.

For this study five scenarios are modeled. The analysis begins with a baseline scenario that assumes the current socio-technical framework remains unchanged. The other scenarios involve several altered factors and spatial implications. The scenarios are distinguished as follows: 1. *Baseline*, 2. *Wind Efficiency & Demand Sufficiency*, 3. *Wind Power Density*, 4. *Wind Efficiency & Wind Power Density* and 5. *Nature Conservation Minus*.

The *Baseline* scenario reflects the current conditions, using the study region’s calculated energy demand, *Enercon E-138 EP3* turbines, and *Canadian Solar HiKu6* PV modules. Wind and PV sites are allocated beginning with the most efficient locations. In *Wind Efficiency & Demand Sufficiency*, energy demand is halved over the modeling period, and a more efficient turbine, the *Enercon E-175 EP5 E1*, is used, requiring greater spacing due to its larger rotor. Technological advances in industry and the economy could reduce future energy demand. So could a change in the population’s energy awareness. The *Wind Power Density* scenario tests the effects of halving the legally mandated minimum distance between wind turbines and residential areas. *Wind Efficiency & Wind Power Density* combines the *E-175 EP5* turbine with this reduced setback requirement. In the *Nature Conservation Minus (NCM)* scenario, nature conservation constraints are relaxed, increasing the land available for RE deployment. Table 3 summarizes these scenarios.

**Table 3:** Overview of the scenarios applied with the used wind power plant (WPP) and photovoltaic plant (PVP) models

Land use scenario	Description
Baseline	WPP: *Enercon E-138 EP3*; PVP: *CanadianSolar HiKu6 Mono PERC*; energy demand as calculated.
Wind Efficiency & Demand Sufficiency	WPP: *Enercon E-175 EP5*; PVP: *CanadianSolar HiKu6 Mono PERC*; energy demand halved.
Wind Power Density	WPP: *Enercon E-138 EP3*; PVP: *CanadianSolar HiKu6 Mono PERC*; energy demand as calculated; the legally required minimum WPP distance to all surfaces is reduced by half; distribution of the PV sites across the entire region.
Wind Efficiency & Wind Power Density	WPP: *Enercon E-175 EP5*; PVP: *CanadianSolar HiKu6 Mono PERC*; energy demand as calculated; the legally required minimum WPP distance to all surfaces is reduced by half; distribution of the PV sites across the entire region.
Nature Conservation Minus	WPP: *Enercon E-138 EP3*; PVP: *CanadianSolar HiKu6 Mono PERC*; energy demand as calculated; reduced nature conservation efforts increasing the available land for RE sites.

### Siting algorithm

The siting algorithm was created with the R programming environment.

The R script operates by first identifying suitable sites for wind and PV, prioritizing wind because of its higher yield per hectare. For reasons of profitability, all locations with wind speeds below 5.5 m/s at hub height are initially excluded. Only above this wind speed the turbines can be operated economically (Energieatlas Bayern 2025). Potential wind sites are ranked by estimated yield and distance ellipses, aligned with the dominant southwesterly wind, are generated around each site. The ellipses have a major axis five times the rotor diameter and a minor axis three times the diameter (FA Wind 2019). Overlapping ellipses are analyzed, and any ellipse whose core overlaps with that of a higher-yield site is removed to maintain proper turbine spacing. Remaining sites are then evaluated against regional wind-energy needs, and the annual number of turbines required is calculated. In the final year, when wind cannot fully meet demand, all remaining wind sites are used and the unmet demand is shifted to PV. For all years without remaining wind potential, the full demand is assigned to PV, triggering a *Switch* from wind to PV once wind resources are exhausted. After allocating wind sites, PV deployment is modeled. Sites are first ranked by potential, then annual PV and remaining *Switch* requirements are calculated. PV plants are assigned accordingly. A final dataset is produced containing the coordinates, installation year, and yield of each wind turbine and PV system, which is then returned to the GIS for visualization.

## Results

In the following, the cartographic and quantitative outputs of the different energy futures are presented.

### Baseline

**Fig. 1:**
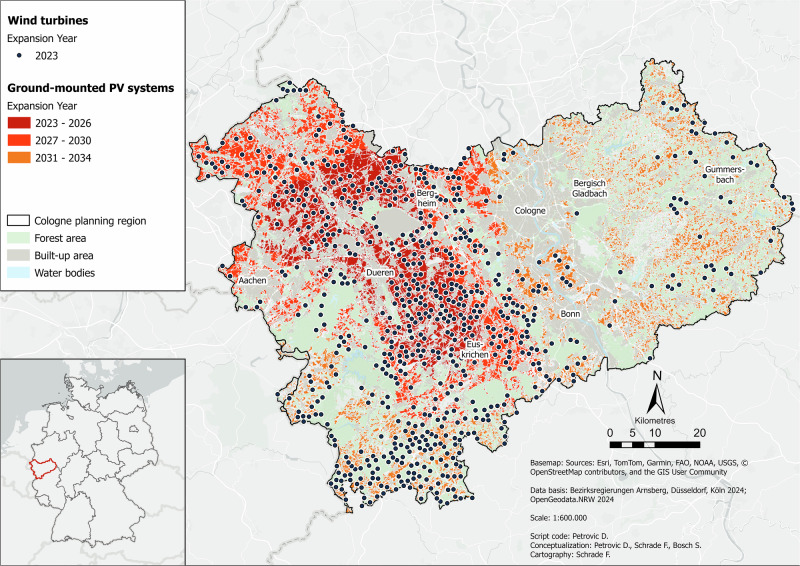
Baseline scenario for the Cologne administrative district in the year 2045. Data basis: Bezirksregierungen Arnsberg, Düsseldorf, Köln (2024); OpenGeodata.NRW (2024)

Figure 1 displays what a future, more climate-neutral energy system could look like in the Cologne administrative district under the current planning framework. The spatial and temporal distributions of the different energy technologies over the modeled period are shown.

In this scenario, large parts of the region are covered with wind turbines. There is a clear contrast between the eastern part of the region and the rest. While the eastern part is only sparsely covered with turbines, there are several dense turbine clusters in the rest of the region. The cluster in the center is the most pronounced, followed by those in the north and south. The region’s existing wind potential is not even sufficient to cover the energy demand for the first year (2023). This illustrates the current, highly limiting conditions. As a result, the *Switch* will already begin in the first year, which means that the combined potential of wind and PV will only last until 2034, ten years less than the target date of 2044. In this *Baseline* scenario, wind turbines and PV systems are often located directly next to each other. PV systems are also concentrated mainly in the western part of the region, with some very pronounced clusters. These correspond well with the wind turbine clusters. This high degree of correspondence indicates very large exclusion areas for both technologies. This is also reflected in the fact that a relatively large area is completely free of both technologies. In the east of the region, PV systems are more scattered and less concentrated. The PV clusters in the western area in the center and northeast are the PV locations with the highest potential, as these will be occupied first.

In this scenario, the existing wind potential covers only 8% of wind energy demand. Overall, i.e., after the *Switch*, 77% of RE demand will be covered. The total number of wind turbines here is 671, which corresponds to a space requirement of 335.5 ha and a total capacity of 2.86 GW. The number of PV systems installed over the entire period is 163,730, which corresponds to an equally large area requirement in hectares and a total capacity of 688 GWp.

### Wind Efficiency & Demand Sufficiency

**Fig. 2:**
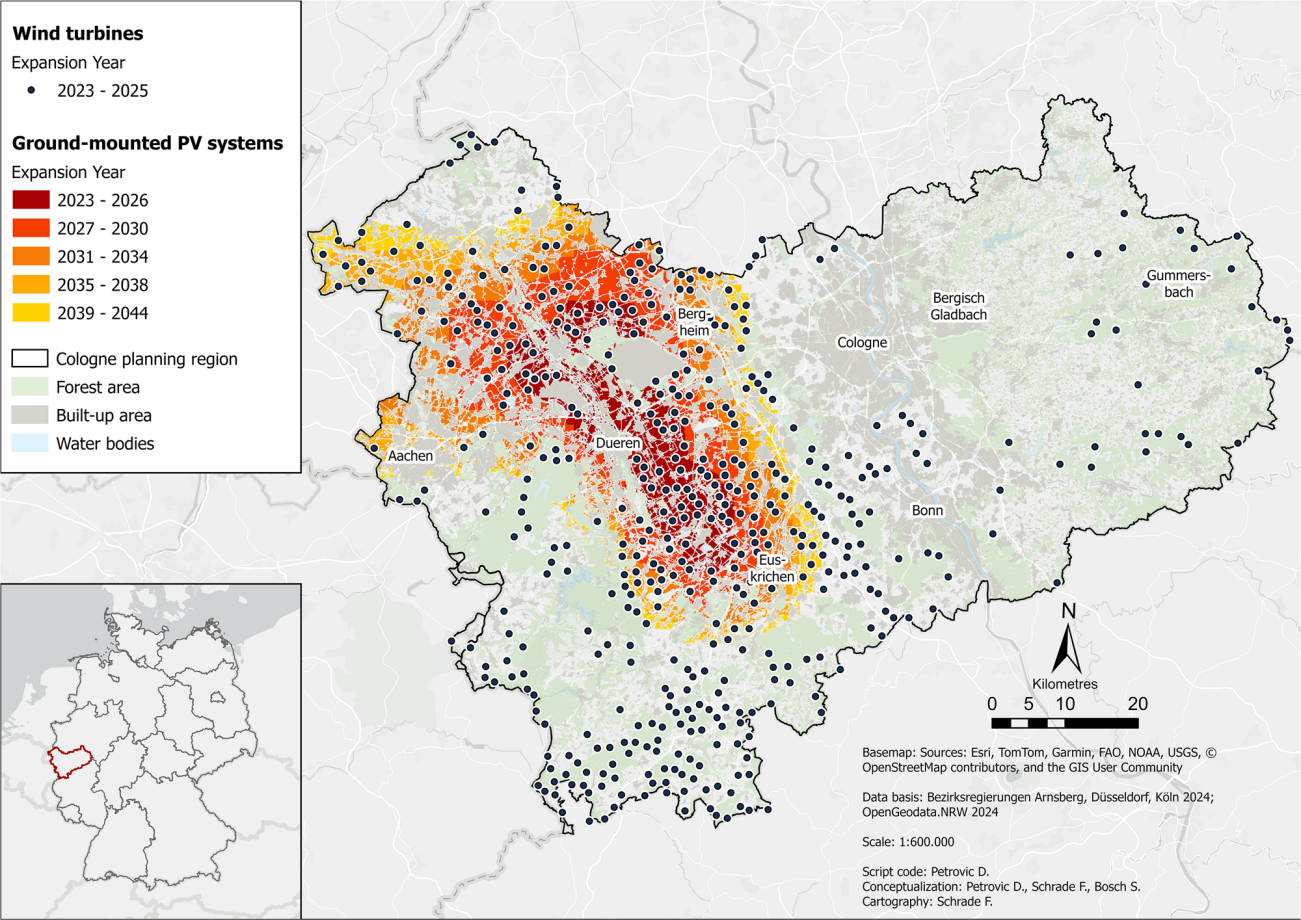
Wind Efficiency & Demand Sufficiency scenario for the Cologne administrative district in the year 2045. Data basis: Bezirksregierungen Arnsberg, Düsseldorf, Köln (2024); OpenGeodata.NRW (2024)

Figure 2 shows the effects of using more efficient wind turbines, combined with more optimized energy use.

The biggest difference compared to *Baseline* is obviously that in this scenario, the RE potential is sufficient until the end of the period in 2044. Energy demand is mainly covered by PV. The wind potential here lasts until 2025 before the *Switch* starts, which is two years longer than in *Baseline*. This is not a very big change and highlights the region’s lack of wind capacity. It also shows that the assumed halving of energy demand is decisive for the changes compared to *Baseline*, not the more efficient wind turbines. The wind locations are basically similar to those in *Baseline*. Overall, there are slightly fewer sites due to the larger wind turbines and the associated greater distances required. The wind turbine clusters from *Baseline* (mainly in the center between Euskirchen and Düren, in the north and south) are also visible here. They are slightly less dense due to the higher spacing rules. In this scenario, too, the east of the region is very sparsely covered with wind turbines. Here, too, wind turbines and PV systems are often located very close to each other. Overall, there are significantly fewer PV systems in this scenario than in *Baseline*. These are highly concentrated from the center to the northwest, while the rest of the region is almost free of PV systems. In the PV cluster, it is clearly visible that sites were occupied from the inside out in chronological order. This means that the inner locations are those with the higher yield potential. In this scenario, large parts of the region are completely free of RE technologies, especially in the east, southwest, and far north.

The covered wind energy demand in this scenario is 18%. Overall, 100% of energy demand will be covered after the *Switch* in 2024. The number of wind turbines is 458, which corresponds to an area of 229 ha. There are 97,093 PV systems in this scenario, which corresponds to an equally high space requirement in hectares. The installed wind capacity here is 2.75 GW and the installed PV capacity is 408 GWp.

### Wind Power Density

**Fig. 3:**
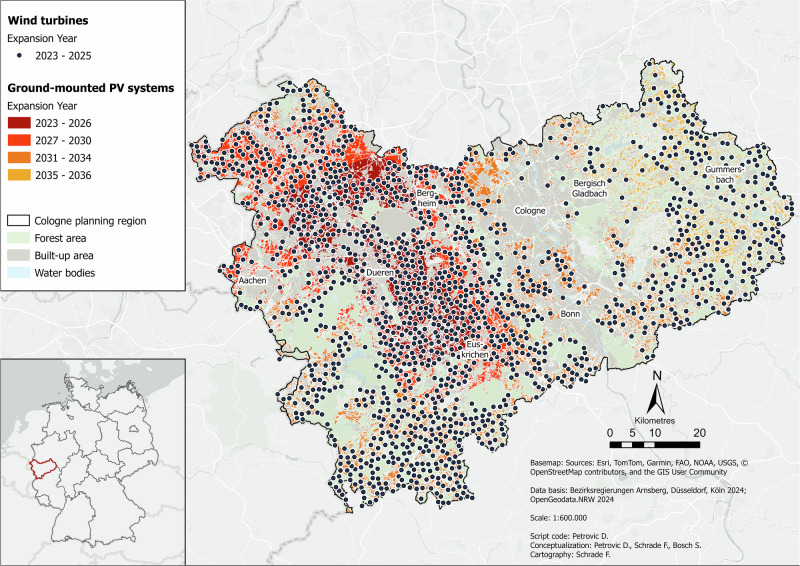
Wind Power Density scenario for the Cologne administrative district in the year 2045. Data basis: Bezirksregierungen Arnsberg, Düsseldorf, Köln (2024); OpenGeodata.NRW (2024)

Figure 3 illustrates how the distribution of RE sites would change if the legal setback requirements for wind turbines were halved. This would enable significantly more turbines to be installed.

Compared to *Baseline*, the existing energy potential here will last two years longer, i.e., until 2036 instead of 2034. Wind energy will last two years longer in this scenario (2025 instead of 2023). In direct comparison to *Baseline*, it becomes clear that significantly more wind turbines are installed here due to the changed regulations, i.e., the minimum distances have been halved. The clusters from *Baseline* are even more pronounced here. Many areas that were previously free of wind power are now occupied. This is particularly evident in the east of the region. Of course, this is associated with very large interventions in the landscape. Since all potential locations for wind and PV are also required in this scenario, the PV distribution here is identical to that of *Baseline*. Only the timing of the occupation varies in some cases. This mainly affects the locations in the northern area, which suggests that these are the locations with the lowest PV potential, as they are occupied last.

In this scenario, 17% of wind energy demand is covered. Overall coverage amounts to 82%. A total of 1550 wind turbines are installed, requiring an area of 775 ha. The total installed wind capacity is 6.6 GW. The number of PV systems and the area occupied is 163,129 (ha), which corresponds to a total capacity of 685 GWp.

### Wind Efficiency & Wind Power Density

**Fig. 4:**
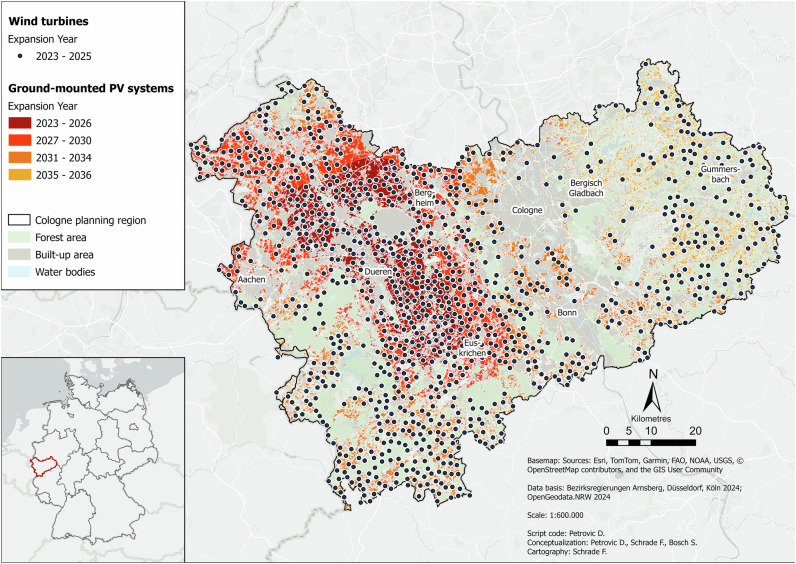
Wind Efficiency & Wind Power Density scenario for the Cologne administrative district in the year 2045. Data basis: Bezirksregierungen Arnsberg, Düsseldorf, Köln (2024); OpenGeodata.NRW (2024)

Figure 4 displays the combined effects of using more efficient wind turbines alongside halved legal setback requirements.

In this scenario, significantly more wind turbines are installed compared to *Baseline*, especially in the north and east. However, in direct comparison to the previous scenario, there are fewer turbines, which is due to the larger dimensions of the systems. The existing turbine clusters from *Baseline* are also reinforced here, albeit not as significantly as in the previous scenario. This is also related to the larger turbines. As in the previous scenario, the wind potential here only lasts until 2025 and the total energy potential until 2036. This means that there is not enough potential in this scenario either. Here, too, all potential PV sites must be used, so the distribution is identical to *Baseline*. In terms of installation dates, there are very strong similarities with the previous scenario. Even though fewer wind turbines are installed here than in the previous scenario, the impact on the landscape remains immense.

In this scenario, wind energy covers 18% of demand, with total RE coverage at 83%. A total of 1033 wind turbines is installed, covering an area of 516.5 ha and with a capacity of 6.2 GW. The number of PV systems and the area occupied is 163,482 (ha), which corresponds to a total capacity of 687 GWp.

### Nature Conservation Minus (NCM)

**Fig. 5:**
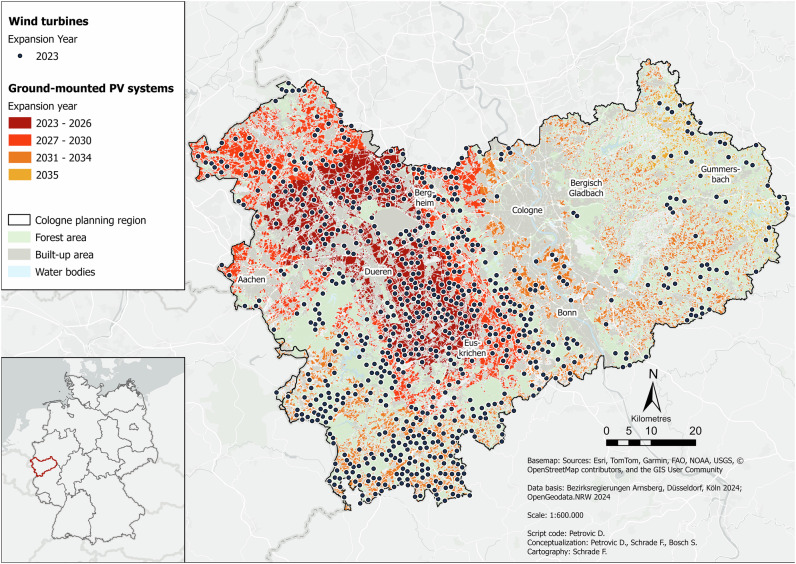
Nature Conservation Minus scenario for the Cologne administrative district in the year 2045. Data basis: Bezirksregierungen Arnsberg, Düsseldorf, Köln (2024); OpenGeodata.NRW (2024)

Figure 5 shows the *NCM* scenario, which simulates the effects of removing conservation restrictions.

In this scenario, wind potential also only lasts for the first year as in the *Baseline* scenario. Overall, RE potential lasts until 2035, which is one year longer than in *Baseline*. This means that the relaxation of nature conservation regulations has only a limited effect. Additional wind locations are mainly located around the region’s center, with the contrast being particularly high in the southern area. Since more areas are available for wind power here than in *Baseline*, there are differences in the distribution of PV. However, it is similar. In the east, very few wind turbines will be installed.

In this scenario, 9% of wind energy demand and a total of 80% of RE demand is covered. Here, 758 wind turbines are installed, requiring an area of 379 ha. There are 169,439 PV systems, also requiring the same amount of hectares. The installed wind capacity is 3.23 GW and the installed PV capacity is 712 GWp.

## Discussion

### Comparison of the scenarios

The results show that climate-neutral electricity production in the Cologne administrative district is achievable only in the *Wind Efficiency & Demand Sufficiency* scenario; all other scenarios fall short. This is one of the key findings of this study. Consequently, none scenario can reach the targeted 50/50 wind-PV distribution. A central finding is the severe shortage of land suitable for wind energy, indicating that current planning rules are inadequate for meeting regional energy transition goals. The success of the *Wind Efficiency & Demand Sufficiency* scenario is primarily due to its reduced energy demand assumptions. Wiese et al. (2022), who conducted a meta-analysis of studies on key strategies for the energy transition in Germany, emphasize that reducing energy demand is one of the most important levers for achieving the goal of climate neutrality.

Table 4 provides an overview of the various degrees of energy demand coverage, both overall and for wind power alone, with the corresponding annual figures showing how long the respective potential will suffice, depending on the scenario. As mentioned above, only the *Wind Efficiency & Demand Sufficiency* scenario achieves 100% coverage of total demand, while the gaps in all other scenarios are considerable. In the *Baseline* scenario, i.e., with the current framework conditions, the lowest total coverage rate of 77% is achieved, and the total potential is also sufficient for the shortest period (until 2035). This is closely followed by the *NCM* scenario, with a total coverage rate of 80%. This illustrates that reduced nature conservation in this region would only have a limited effect. As mentioned above, the total coverage of 100% of energy demand and the coverage of the entire period up to 2044 in the *Wind Efficiency & Demand Sufficiency* scenario is mainly due to the halving of energy demand. This is particularly evident in the fact that wind coverage has not really increased. In the scenarios *Wind Power Density* (17%) and *Wind Efficiency & Wind Power Density* (18%), it is roughly the same. The values from *Wind Power Density* and *Wind Efficiency & Wind Power Density* are very similar, both in terms of total coverage and wind coverage. Looking specifically at wind coverage, the differences in annual expansions are not very large, with a maximum difference of two years between 2023 and 2025. This illustrates that the possibilities for providing more wind areas are very limited in this region. In the scenarios *Wind Efficiency* & *Demand Sufficiency, Wind Power Density* and *Wind Efficiency* & *Wind Power Density*, the wind potential lasts the longest, namely until 2025. These three scenarios also achieve the highest wind coverage rates of 17% and 18%, respectively. This means that, in the best-case scenarios, less than one-fifth of the region’s wind energy demand can be covered. The lowest coverage rates are achieved in the *Baseline* and *NCM* scenarios (8% and 9%, respectively). This once again illustrates the limited effect of reduced nature conservation on the approval of more wind areas. Since none of the scenarios even come close to achieving 100% demand coverage from wind power, every single turbine is needed in each scenario.

**Table 4:** Overview of the degrees of energy demand coverage overall and for wind specifically with the corresponding last years of possible coverage

Land use scenario	Last covered year overall	Overall demand coverage [%]	Last year of wind coverage	Wind demand coverage [%]
Baseline	2034	77	2023	8
Wind Efficiency & Demand Sufficiency	2044	100	2025	18
Wind Power Density	2036	82	2025	17
Wind Efficiency & Wind Power Density	2036	82	2025	18
Nature Conservation Minus (NCM)	2035	80	2023	9

**Fig. 6:**
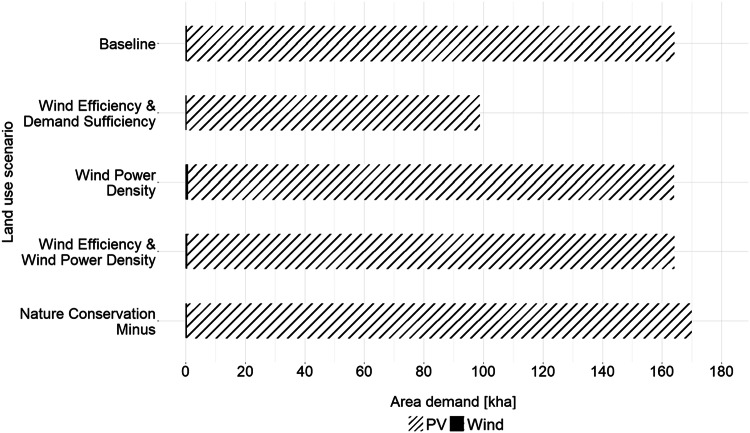
Graphical representation of the space requirements

Figure 6 visualizes the space requirements for PV and wind in the individual scenarios. It is clear that, in terms of pure space, wind plays only a minor role in each scenario. The PV areas are several orders of magnitude larger in all scenarios. This is because significantly more PV areas are available in all scenarios except *Wind Efficiency & Demand Sufficiency*, and all of these are also needed. The total space requirements between scenarios *Baseline*, *Wind Power Density*, and *Wind Efficiency & Wind Power Density* are quite similar, all about 164,000 ha. The great similarity between the *Wind Power Density* and *Wind Efficiency & Wind Power Density* scenarios, both in terms of coverage (Table 4) and space requirements (Fig. 6), suggests that the more efficient wind turbines in *Wind Efficiency & Wind Power Density* do not have a really significant impact. In fact, the greater spacing requirements resulting from the larger rotors of the more efficient turbine mean that there is less wind space overall in *Wind Efficiency & Wind Power Density* compared to *Wind Power Density*. As mentioned above and as can be inferred from Fig. 6, the wind area in *Wind Power Density* is 775 ha and thus higher than the 516.5 ha in *Wind Efficiency & Wind Power Density*. The space requirement in the *NCM* scenario is slightly higher, at a total of around 170,000 ha. This means that relaxed nature conservation leads to an increase of around 6,000 ha in RE space. The lowest land requirement is naturally found in the *Wind Efficiency & Demand Sufficiency* scenario, with a total of approximately 99,000 ha, which is considerably less than in the other scenarios. In all cases, the expansion of RE systems with the corresponding land requirements would involve immense changes to the landscape. The type of reaction that such a far-reaching change would provoke is unclear, but previous studies suggest that resistance from the local population is to be expected (e.g., Bauwens et al. 2016; Reusswig et al. 2016; Giordono et al. 2018). The reasons for this are manifold and include, for example concerns about changes to scenic landscapes (Gkeka-Serpetsidaki and Tsoutsos 2023), impacts on biodiversity (Thaxter et al. 2017), ecosystems (Galparsoro et al. 2022), human health (Bakhiyi et al. 2014; van Kamp and van den Berg 2021) and local economies (McKenna et al. 2025).

### Spatial injustices

Another factor besides the impact on the landscape is the spatial concentration of RE systems. In the *Baseline* scenario, the region as a whole is highly polarized due to the stark contrast between west and east, especially in terms of wind power coverage. This could lead to tensions among local populations, as it may be perceived as unfair in those places with high concentrations and the associated high level of intervention. According to Yenneti et al. (2016), this can also be referred to as spatial “injustices”, where smaller parts of a region bear the main load of large-scale landscape interventions for energy production, while the rest of the region experiences few or no constraints. This applies to virtually all scenarios except, to a lesser extent, to those with greater wind density, i.e., *Wind Power Density* and *Wind Efficiency & Wind Power Density*, as concentrations and thus spatial-technological polarization occur everywhere. It can be assumed that a distribution of wind turbines like in these two scenarios would stimulate strong opposition from local populations due to the large number of turbines and the associated impact on the landscape. Nevertheless, these scenarios would be somewhat fairer than the others, as the scenic burdens caused by the turbines would be distributed more evenly across the region, meaning that a much larger part of the region would contribute to the energy transition. This would also be a form of energy justice. When discussing spatial injustices or inequalities, it is important to remember that their meaning can be interpreted differently depending on the context and the actors involved (Yenneti et al. 2016; Bailey and Darkal 2018; Cha 2020; Urkidi Azkarraga and Gurrutxaga 2024). One way to overcome these spatial injustices is through financial compensation. This means that areas or communities with a high concentration of RE systems could generate income by exporting surplus energy to areas or communities without local production. From this income the local population could benefit (see, for example, Parkins et al. 2022; van Wijk et al. 2021; Lindvall 2023). Juntunen and Martiskainen (2021) emphasize that local energy production could also bring autonomy benefits, as the affected areas or communities would become less dependent on energy imports. Furthermore, Busch and McCormick (2014) cite other potential benefits of local energy production. On the one hand, there are economic advantages in the form of more jobs and income and the associated higher tax revenues, from which in turn the population could benefit. On the other hand, there are social benefits such as strengthening the community and experiencing progress. In a democratic country like Germany in particular, the local level is crucial for the acceptance and success of the energy transition, as resistance here can quickly block the implementation of new projects.

### Spatial boundaries (of climate neutrality) and external options

Climate-neutral electricity production is not feasible in most scenarios for the Cologne administrative district. The region’s large population of about 4.5 million people (Statistisches Landesamt NRW 2023) and its resulting high energy demand plays a major role. Urban centers such as Aachen, Bonn, Leverkusen, and especially Cologne intensify this demand. In addition, the district’s diverse and energy-intensive industries like insurance, media, mechanical engineering, electrical engineering, chemical and pharmaceutical industries further increase consumption, even though this is only partly captured in population-based calculations. Another reason are the strict planning restrictions, as has become apparent. This also limits the availability of suitable sites for RE, making it unlikely that enough land can be provided. Nevertheless, through a series of measures, largely climate-neutral electricity consumption remains possible in the district. One of the most effective levers is a significant reduction in energy demand, as this study has shown (*Wind Efficiency & Demand Sufficiency* scenario). This could be achieved through efficiency measures in buildings, industry, and transportation. Examples include insulation, efficient motors, heat pumps, e-mobility and waste-heat use. In addition, advanced heat and battery storage systems can play a crucial role in ensuring that the available renewable electricity is used optimally (Agora Energiewende, Prognos, Consentec 2022). As already mentioned, remaining demand could be met by importing green electricity from surplus regions (FfE, Consentec 2024). Smart grids could help reduce bottlenecks through better control of electricity flows (Agora Energiewende, Prognos, Consentec 2022). Climate-neutral fuels such as green hydrogen used, for example, in hydrogen-ready or synthetic fuel gas plants, could help cover residual loads, with imports supplementing local production (Agora Energiewende, Prognos, Consentec 2022). Hard-to-avoid emissions can be offset through high-quality CO₂ certificates or climate protection projects, with focus on genuine emission reduction and avoidance (GermanZero e.V. 2025). Finally, it is important to use as much of the existing potential locally as possible. This includes, for example, the use of rooftop and facade PV, waste-based biomass, geothermal energy, and hydropower (BEE 2025b).

Against this background, it should be emphasized that this analysis is confined to the Cologne planning region, representing a meso-level scale between the municipal (micro) and national (macro) levels. The chosen spatial boundary significantly shapes the results. Energy systems operate across administrative borders, and complete regional self-sufficiency is often neither realistic nor necessarily desirable, although it may be achievable under specific structural conditions. While broader integration at the macro level enables balancing effects between regions with different renewable potentials, local conditions at smaller scales may lead to different outcomes. The findings must therefore be understood as scale-specific and cannot be directly transferred to other spatial levels.

### Limitations of the study

This study has several limitations. It does not account for energy storage or weather-related off-times (e.g., Suh et al. 2017; Liu et al. 2018; Hou et al. 2020; Zhang et al. 2020), both of which would reduce usable generation capacity. The same applies to shutdown periods for species protection. These are primarily intended to protect birds and bats (e.g., FA Wind 2018; BfN 2020; KNE 2025). This aspect is not considered here either. However, the omission does not affect the main conclusion that there is not enough space for climate-neutral energy transition, except in the *Wind Efficiency & Demand Sufficiency* scenario, where storage could slightly reduce required capacity. The analysis also does not fully reflect system-level costs such as grid and storage expansion (Lehmann et al. 2023), and it assumes constant technologies throughout the modeling period. Current and potential future technical developments are therefore not shown here. One example of this is the use of high-altitude wind turbines. Such a turbine is currently under construction in Brandenburg, Germany. With a hub height of 300 m, it will be the tallest wind turbine in the world (e.g., GICON 2025). Such turbines could achieve higher yields. Nevertheless, this does not affect the core messages of this study. Although future turbines or PV systems may become more efficient, the technological leaps required to offset the land shortage are unrealistic. Repowering is likewise excluded but would not change the core findings. Political shifts or unforeseen events such as the Russian invasion of Ukraine or, as is currently being discussed again, the end of combustion engines in Germany, which is actually scheduled for 2035, could alter future energy targets, but these cannot be anticipated here. Economic viability is also not assessed, as the study focuses solely on siting potential. Generally, it is difficult for us to assess economic efficiency due to a lack of available data and information. Lastly, it should be noted that the quality of our modeling depends strongly on the accessibility and accuracy of the geospatial data used as input. Certain areas, including those of military security significance and for which geospatial data is unavailable, are excluded from the scope of our study. This could have an impact on the availability of land for RE energy systems. However, we do not assume that this impact will be substantial enough to modify the overall conclusions.

## Conclusions

Germany has committed to becoming climate neutral by 2045. The energy transition is crucial to achieving this goal. This requires a massive expansion of RE systems, especially wind and PV. This expansion must be significantly accelerated, as the measures taken so far are not sufficient. A key challenge for Germany is the availability of land for RE systems due to numerous spatial restrictions. In this study, the expansion of RE systems up to 2045 was carried out taking into account the Paris climate targets, using the administrative district of Cologne as an example, a region with a high population number and density, a strong economy and industry, and diverse land uses and restrictions. By varying planning regulations in different scenarios, various energy landscape options were modeled.

The results reveal that climate-neutral electricity production is not possible in the Cologne administrative district under the current planning law, as there is insufficient space available for PV and wind power. Even changes to the planning law such as reducing the minimum distances between wind turbines and lowering nature conservation standards, are not sufficient to create the required capacity. These measures would only help to extend the period that can be covered by wind and PV energy. Only a significant reduction in energy demand –assumed to be 50% in this study – would have such a major impact that electricity production could be climate-neutral by 2045. The results also show that, in every scenario, the energy transition would be associated with major changes to the landscape due to the large amount of space required for the RE systems. The extent of these changes varies depending on the scenario.

The study showed that combining a GIS-based approach with a siting algorithm can be a powerful tool for modeling different energy landscape options within spatial constraints to achieve climate-neutral electricity production.

## Data Availability

Data will be made available on reasonable request.
